# Decreased Intracranial Pressure Elevation and Cerebrospinal Fluid Outflow Resistance: A Potential Mechanism of Hypothermia Cerebroprotection Following Experimental Stroke

**DOI:** 10.3390/brainsci11121589

**Published:** 2021-11-30

**Authors:** Daniel Omileke, Steven W. Bothwell, Debbie Pepperall, Daniel J. Beard, Kirsten Coupland, Adjanie Patabendige, Neil J. Spratt

**Affiliations:** 1 School of Biomedical Sciences and Pharmacy, The University of Newcastle, Callaghan, NSW 2308, Australia; daniel.omileke@uon.edu.au (D.O.); steven.bothwell@uon.edu.au (S.W.B.); debbie-gai.pepperall@newcastle.edu.au (D.P.); daniel.j.beard@newcastle.edu.au (D.J.B.); kirsten.coupland@newcastle.edu.au (K.C.); 2 Hunter Medical Research Institute, New Lambton Heights, Newcastle, NSW 2305, Australia; 3 Institute of Infection, Veterinary & Ecological Sciences, University of Liverpool, Wirral CH64 7TE, UK; 4 Department of Biology, Edge Hill University, Ormskirk L39 4QP, UK; 5 Hunter New England Local Health District, New Lambton Heights, Newcastle, NSW 2305, Australia

**Keywords:** hypothermia, cerebrospinal fluid, outflow resistance, ischaemia, intracranial pressure, stroke

## Abstract

Background: Elevated intracranial pressure (ICP) occurs 18–24 h after ischaemic stroke and is implicated as a potential cause of early neurological deterioration. Increased resistance to cerebrospinal fluid (CSF) outflow after ischaemic stroke is a proposed mechanism for ICP elevation. Ultra-short duration hypothermia prevents ICP elevation 24 h post-stroke in rats. We aimed to determine whether hypothermia would reduce CSF outflow resistance post-stroke. Methods: Transient middle cerebral artery occlusion was performed, followed by gradual cooling to 33 °C. At 18 h post-stroke, CSF outflow resistance was measured using a steady-state infusion method. Results: Hypothermia to 33 °C prevented ICP elevation 18 h post-stroke (hypothermia ∆ICP = 0.8 ± 3.6 mmHg vs. normothermia ∆ICP = 4.4 ± 2.0 mmHg, *p* = 0.04) and reduced infarct volume 24 h post-stroke (hypothermia = 78.6 ± 21.3 mm^3^ vs. normothermia = 108.1 ± 17.8 mm^3^; *p* = 0.01). Hypothermia to 33 °C did not result in a significant reduction in CSF outflow resistance compared with normothermia controls (0.32 ± 0.36 mmHg/µL/min vs. 1.07 ± 0.99 mmHg/µL/min, *p =* 0.06). Conclusions: Hypothermia treatment was protective in terms of ICP rise prevention, infarct volume reduction, and may be implicated in CSF outflow resistance post-stroke. Further investigations are warranted to elucidate the mechanisms of ICP elevation and hypothermia treatment.

## 1. Introduction

We have previously demonstrated that there is a damaging, transient increase in intracranial pressure (ICP) occurring 18–24 h post-stroke in rats [1,2,3,4,5,6]. Evidence suggests that ICP elevation has detrimental consequences on stroke outcome and may cause delayed expansion of the infarct core into the penumbral region. Penumbral tissue is maintained by perfusion from the leptomeningeal collaterals following stroke [7,8,9]. The disruption of autoregulatory mechanisms during stroke results in cerebral perfusion pressure (CPP) becoming the driving force of this collateral perfusion. ICP is directly related to CPP as CPP is the difference in pressure between ICP and mean arterial pressure. We have shown that an increase in ICP causes a reduction in CPP and results in reduced flow through the leptomeningeal collateral vessels [3,10]. Short-duration hypothermia is a potential therapy and has previously been shown to prevent this elevated pressure following experimental stroke [1,2,4]. Our understanding of the mechanism of ICP elevation is incomplete, thus the mechanism of how hypothermia works to prevent ICP elevation is not clear and warrants investigation.

The Monroe–Kellie doctrine [11,12] describes ICP as the pressure that exists within the craniospinal compartment, and states that this compartment is a closed system which comprises a fixed volume. This volume is composed of blood, brain tissue and CSF within the skull. Normally, the system can compensate when there is an increase in the volume of one component of the compartment by decreasing the volume of one of the other components. However, when this compensatory mechanism fails, it results in increases in ICP. This suggests that the mechanisms responsible for ICP elevation derive from an increase in volume in one or more of the components of the craniospinal compartment. Our group have previously demonstrated that the dramatic increases in ICP observed at 24 h in rats following ischaemic stroke is not primarily driven by oedema [2]. Additionally, unpublished data from our group have also shown that increased cerebral blood volume does not contribute to ICP elevation post-stroke. These findings suggest that CSF volume is the likely driver of ICP elevation, and this observation has been backed by preliminary data from our group [6] and reproduced by others [13].

CSF volume can be altered in the craniospinal space by two pathways; increased CSF production and decreased CSF outflow, both of which would result in an overall increase in the volume of CSF within the compartment. CSF volume is very difficult to quantify in vivo but we are able to determine insights into CSF dynamics by measuring CSF production or CSF outflow resistance (resistance to the efflux of CSF out of the craniospinal compartment) in real time. Previous work from our group has demonstrated that there is an increase in CSF outflow resistance in rats subjected to cortical stroke compared with sham rats 18 h post-stroke [6]. A recent study by Alshuhri et al., using an indirect estimation of CSF production at 24 h post-stroke, could not detect an increase in CSF production in stroke animals compared with sham [13]. These results suggest that CSF outflow resistance may be an important component that contributes to increased CSF volume, and therefore is implicated in ICP elevation.

We have previously demonstrated that ultra-short duration hypothermia, using clinically relevant cooling rates, prevents ICP elevation and reduces infarct volume 24 h post-stroke in rats [14]. If the main driver of ICP elevation post-stroke is due to an increase in CSF outflow resistance, it is plausible that hypothermia would decrease CSF outflow resistance and lead to reduced ICP elevation post-stroke in hypothermia animals compared to normothermia controls. We aimed to investigate whether ultra-short duration hypothermia using clinically relevant cooling rates reduces CSF outflow resistance 18 h following experimental stroke, when compared with normothermia controls.

## 2. Materials and Methods

### 2.1. Animals

Adult male (11–12 weeks old) outbred Wistar rats (*n* = 32, Animal Services Unit, University of Newcastle, Callaghan, Australia) weighing 280–350 g were used for this study. Animals were housed under standard conditions in a 12 h light-dark cycle with unlimited access to food and water. All experimental procedures were in accordance with the Australian Code of Practice for the Care and Use of Animals for Scientific Purposes and were approved by the Animal Care and Ethics Committee of the University of Newcastle (A-2013-343 and A-2020-003).

### 2.2. Anaesthesia and Physiological Monitoring

Rats were anesthetized with isoflurane (5% induction, 2–2.5% maintenance) in 50:50 N_2_:O_2_. Incision sites were cleaned and injected subcutaneously (s.c.) with 2 mg/kg 0.05% Bupivacaine (Pfizer, Sydney, Australia). Body temperature was regulated throughout the surgery with a rectal temperature thermocouple (RET-2, Physitemp Instruments Inc., Clifton, NJ, USA). The femoral artery was cannulated with a catheter (1 and 2 French silicone tubing) for continuous monitoring of arterial blood pressure and heart rate. After stroke surgery and hypothermia treatment, rectal paracetamol (250 mg/kg, GlaxoSmithKline, Brentford, UK) was administered for overnight pain relief. Saline injections (2 × 1.5 mL, s.c.) were also given to prevent dehydration and animals were returned to their cages with free access to softened laboratory chow and water.

### 2.3. Implantation of Datalogger Device

Datalogger implantation was performed for continuous monitoring of core body temperature during experimental protocols and recovery. Implantation was performed according to previously described methods [15]. To summarise, a 2 cm longitudinal skin incision was made in the lateral lower right abdominal quadrant to expose the subcutaneous space at the ventral thigh crease. A subcutaneous pocket was created using haemostats and forceps. The pocket was made large enough to hold the temperature monitoring datalogger (Maxim, San Jose, CA, USA). The pocket was secured by closing the skin incision with 4-0 nylon sutures.

### 2.4. Intracranial Pressure and Laser Doppler Measurement

**Figure 1:**
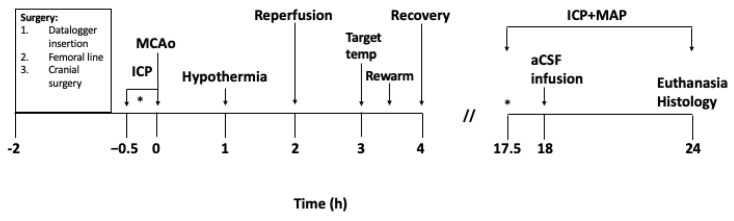
Experimental protocol in hours. MAP = Mean arterial pressure. The asterisk (*) indicates the period of baseline ICP readings for 30 min.

Cranial surgery was performed according to previously described methods [16]. To summarise, the ICP probe (OpSens Fibre Optic Pressure Sensors, Opsens Solutions, Quebec City, QC, Canada) was inserted epidurally into a saline filled, polyether ether ketone (PEEK) screw (Bregma 7 mm posterior and 2 mm lateral) in the left parietal bone. The Laser Doppler probe (Moor Instruments, Devon, UK) was inserted into a second hollow PEEK screw (Bregma 2 mm posterior and 5 mm lateral) in the right parietal bone. Lastly, a third PEEK screw was placed over the lateral ventricle (Bregma 0.8 mm posterior and 2 mm lateral) to be used for infusion of artificial CSF (aCSF). The screws were secured with dental cement and an airtight seal was created around each probe using a caulking material (Silagum, DMG Chemisch-Pharmazeutische, Hamburg, Germany). Correct placement of the ICP probe was confirmed by a response to abdominal compression which was observed on both ICP and arterial blood pressure waveforms. ICP was monitored at pre-stroke baseline and again at 18 h post-stroke: prior to, during and after aCSF infusion until 24 h post-stroke (Figure 1). Tissue perfusion via Laser Doppler flowmetry (LDF) was measured at baseline and throughout the stroke surgery and hypothermia treatment/normothermia. To account for any variation in baseline ICP between groups, change in ICP from baseline to 18 h post stroke (ΔICP) was used for all ICP analyses.

### 2.5. Middle Cerebral Artery Occlusion

Transient middle cerebral artery occlusion (MCAo) was carried out according to our established protocol [17,18]. To summarise, a 7 cm length of monofilament nylon suture with a silicon tip (3 mm length × 0.38 mm O.D silicone) was inserted through the ligated right external carotid artery into the right internal carotid artery. The filament was advanced 20 mm through the internal carotid artery until resistance was felt, and a drop in perfusion units (>50% drop from baseline) on the LDF was observed which indicated that the middle cerebral artery has been occluded. Reperfusion was achieved at 2 h post-MCAo by retracting the monofilament through the internal carotid artery approximately 18 mm until the silicone tip was visible in the external carotid artery stump.

### 2.6. Hypothermia Treatment

We opted for the gradual cooling hypothermia paradigm as opposed to the traditional rapid cooling techniques that are common in pre-clinical literature. This choice was based on previous findings that show that clinically relevant cooling rates prevent ICP elevation and reduce infarct volume [14]. Using this paradigm allows us to make greater assumptions about the clinical translatability of hypothermia and its mechanisms that prevent ICP elevation post-stroke. Animals were randomized to hypothermia treatment or normothermia control immediately after MCAo. Hypothermia was initiated 1 h after vessel occlusion. Animals were cooled gradually at a rate of 2 °C/h to a target core temperature of 33 °C [15]. Once target temperature was reached, animals were maintained at target for 30 min. No external cooling was necessary as anaesthesia prevented normal regulation of core body temperature. For recovery and rewarming, core body temperature was increased to 35 °C prior to recovery from anaesthesia, and animals were placed in a cage half over a warming pad (Passwell, Glen Osmond, Australia) to allow for self-thermoregulation, per our established technique [14]. Animals in the normothermia group were kept at 37 °C for the duration of this period.

### 2.7. Artificial Cerebrospinal Fluid (aCSF) Infusion

CSF outflow resistance was calculated using a steady-state infusion method [19,20] with some modifications. To summarise, a catheter was inserted into the PEEK screw located over the contralateral lateral ventricle. The catheter was inserted 9 mm into the ventricle and secured to the screw using caulking material (Silagum, DMG Chemisch-Pharmazeutische, Hamburg, Germany). Baseline ICP was monitored for 30 min prior to infusion of aCSF (Harvard Apparatus, Holliston, MA, USA), which commenced at 18 h post-stroke. The aCSF infusion was carried out using a syringe driver (Harvard Apparatus, Holliston, MA, USA). The infusion rate was adjusted incrementally, starting from 5 μL/min, then increasing to 10, 15, 20, 25 and 30 μL/min. A 5–10 min period was allowed for a steady-state ICP to be established after each increase. Infusion was stopped once ICP reached 40 mmHg, or after 10 min at 30 μL/min, whichever occurred sooner. This variability in the protocol was necessary to allow for inter-animal differences while also taking animal welfare considerations into account. An investigator blinded to treatment allocation made all decisions relating to the aCSF infusion protocol.

### 2.8. Histological Analysis

Animals were euthanized at 24 h post-stroke. They were transcardially perfused with saline. Brains were removed and sliced into 2 mm sections on a rat brain matrix. Tryphenyltetrazolium chloride (TTC) staining was performed to confirm the presence of ischaemic stroke by identification of infarcted tissue. These same brains were then fixed in neutral-buffered formalin before processing for haematoxylin and eosin (H&E) staining for infarct volume quantification. Images were scanned using a high-resolution scanner (Aperio, Vista, CA, USA) and infarct volumes were analyzed using the Aperio ImageScope program. Infarct analysis was conducted by an independent investigator blinded to treatment allocation. Infarct volumes were corrected for oedema by applying the formula: corrected infarct volume (mm^3^) = infarct volume × (contralateral volume/ipsilateral volume) [2].

### 2.9. Exclusion Criteria and Statistical Analyses

Subarachnoid haemorrhage, equipment malfunction and lack of sufficient LDF drop at occlusion were pre-specified exclusion criteria. For outflow resistance measurements, lack of reliable ICP trace and complications during aCSF infusion that prevented ICP readings from at least 4 infusion rates, were also prespecified exclusions. Sample sizes were based on a similar study [13] which calculated that 6 animals per group were required to detect a 60% difference in CSF outflow resistance between groups, with standard settings of alpha 0.05, power 0.8. Statistical analyses were performed using GraphPad Prism version 9.0. Data were tested for normal distribution using the Shapiro–Wilk normality test. Two-tailed Student’s *t*-test (unpaired) were used to compare differences between hypothermia treatment and normothermia control groups. Relationships were determined by Pearson r correlation. Data are presented as mean ± standard deviation (SD) unless otherwise stated. Statistical significance was accepted at the level of *p* < 0.05.

## 3. Results

A total of 15 animals were included in this study: 9 treated with hypothermia to 33 °C and 6 normothermia controls. A total of 17 animals were excluded. Reasons for exclusion were lack of sufficient LDF drop at occlusion (*n* = 4), abnormal animal physiology (*n* = 1), death during aCSF infusion (*n* = 1), poor oxygenation post-surgical intervention (*n* = 3), subdural bleed during cranial surgery (*n* = 1), surgical error during MCAo (*n* = 1), death at reperfusion with no clear cause detected at post-mortem (*n* = 1), subarachnoid haemorrhage confirmed at post-mortem (*n* = 2), overnight death with no apparent cause detected at post-mortem (*n* = 1), and ICP equipment malfunction (*n* = 2). One animal in the hypothermia group experienced a fever overnight, which may have contributed to an ICP rise in this animal. The animal was included in all analyses, as fever complication was not a pre-specified exclusion criterion. Three animals had infusions stopped before the 30 μL/min infusion rate was reached, because an ICP level of 40 mmHg was reached. These animals were included in all analyses.

### 3.1. Normality Tests

The Shapiro–Wilk normality test indicated that ICP, outflow resistance and infarct volume variables were normally distributed {ΔICP (hypothermia: W = 0.96, *p* = 0.80 and normothermia: W = 0.89, *p* = 0.34), outflow resistance (hypothermia: W = 0.87, *p* = 0.14 and normothermia: W = 0.82, *p* = 0.09) and infarct volume (hypothermia: W = 0.98, *p* = 0.98 and normothermia: W = 0.99, *p* = 0.99)}.

### 3.2. ICP Elevation and Infarct Volume

**Figure 2:**
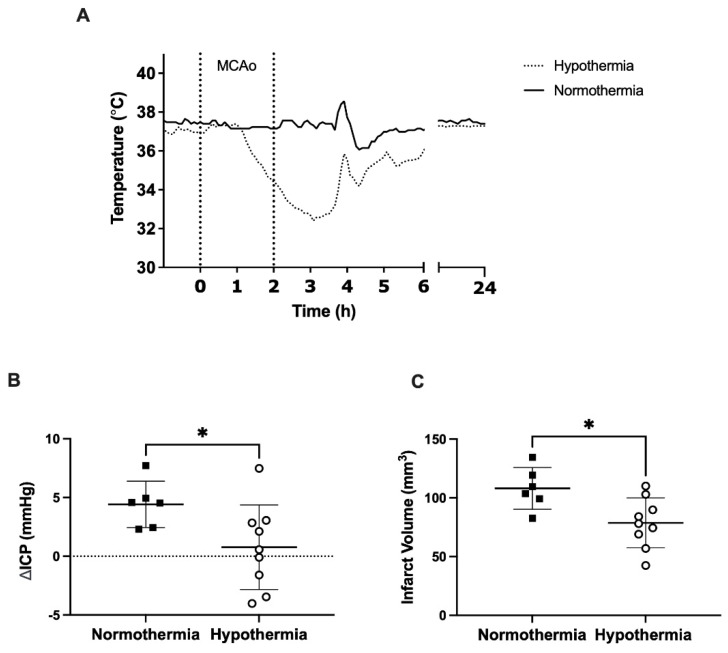
(**A**) Core temperature recordings during hypothermia and equivalent normothermia period. 0–2 h represent period of occlusion. Hypothermia treatment was initiated at 1 h. (**B**) ΔICP from baseline to 18 h in the hypothermia treated and normothermia control animals. (**C**) Infarct volumes from H&E-stained sections at 24 h post-stroke in hypothermia treated and normothermia control animals. * *p* < 0.05.

**Figure 3:**
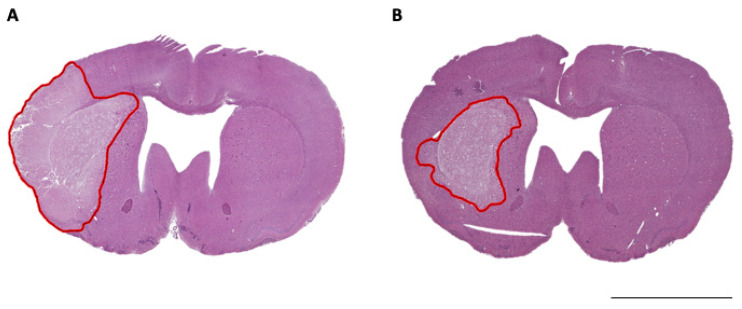
Representative H&E-stained brain sections of (**A**) normothermia and (**B**) hypothermia treated animals. Red line indicates area of infarction. Scale bar = 4 mm.

Accurate temperature regulation was maintained in hypothermia treated animals and normothermia controls (Figure 2A). Mean temperature was 33.0 ± 0.7 °C during hypothermia treatment and 37.2 ± 1.0 °C during the equivalent normothermia period. Target temperature was achieved after a mean of 92.2 ± 31.5 min from hypothermia initiation. ΔICP was significantly higher in the normothermia group vs. the hypothermia treatment group (4.4 ± 2.3 mmHg vs. 0.8 ± 3.6 mmHg, *p* = 0.04: Figure 2B). Normothermia animals had significantly larger infarct volumes compared with hypothermia treated animals (108.1 ± 17.8 mm^3^ vs. 78.6 ± 21.3 mm^3^, *p* = 0.01: Figure 2C). Representative images of infarct size are shown in Figure 3.

### 3.3. CSF Outflow Resistance

**Figure 4:**
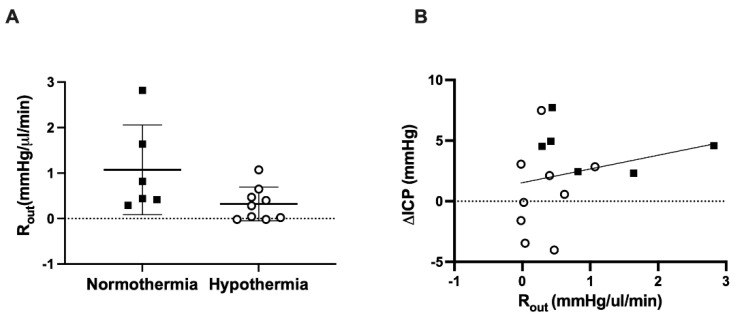
(**A**) R_out_ values between hypothermia treated and normothermia controls. (**B**) Pearson correlation between ΔICP and R_out_ in hypothermia animals (r^2^ = 0.04, *p* = 0.61: white circles) and normothermia animals (r^2^ = 0.09, *p* = 0.56: black squares).

CSF outflow resistance (R_out_) was not significantly different between normothermia controls and hypothermia treated animals 18 h post-stroke (1.1 ± 1.0 mmHg/µL/min vs. 0.3 ± 0.4 mmHg/µL/min, *p =* 0.06: Figure 4A). There was no correlation between outflow resistance and ΔICP at 18 h in hypothermia treated animals (r^2^ = 0.04, *p* = 0.61: Figure 4B) or normothermia control animals (r^2^ = 0.09, *p* = 0.56: Figure 4B).

## 4. Discussion

We have confirmed previous work indicating that clinically achievable gradual cooling effectively prevents ICP elevation and reduces infarct volume post-stroke. We also demonstrated a trend to a physiologically relevant difference in CSF outflow resistance between hypothermia treated animals and normothermia controls 18 h post-stroke. These data suggest that, at least in part, hypothermia cytoprotection may act via reduction of CSF outflow resistance, preventing ICP elevation, and thereby maintaining penumbral perfusion by prevention of failure of leptomeningeal collateral flow.

In our previous study, ICP and infarct volume were measured at 24 h [14]. In this present study, ICP was measured at 18 h post-stroke, prior to aCSF infusion, and infarct volume was assessed at 24 h post-stroke. The earlier ICP time point was necessary to conduct the infusion of aCSF and measure outflow resistance prior to the occurrence of any significant ICP rises. Interestingly, we found that ΔICP at 18 h post-stroke was already significantly greater in normothermia animals compared to hypothermia treated animals. It has been shown that ICP elevation peaks 24 h post-stroke [1,2,4] but recent work by our group has demonstrated significant ICP rises as early as 18 h post-stroke [6,21]. Therefore, these data provide further evidence in favor of hypothermia treatment and its profound effect on ICP, as early as 18 h post-stroke.

Our results suggest that hypothermia treatment may influence CSF outflow post-stroke, with animals treated with hypothermia having lower outflow resistance rates than normothermia controls, albeit not statistically significant. The recent study by Alshuhri et al. [13] demonstrated that there is a significant increase in CSF outflow resistance in stroke rats subjected to permanent MCAo compared to sham control rats. Their data indicated that CSF outflow resistance, rather than oedema, was the mechanism responsible for ICP elevation following ischaemic stroke. We have shown previously that hypothermia results in a sustained prevention of ICP elevation post-stroke [1,2,4]. We have also shown that hypothermia has a powerful effect on infarct volume reduction [1,2]. The results obtained in this present study suggest that outflow resistance may be a mechanism involved in ICP elevation, and that hypothermia may act on this mechanism. The difference obtained in R_out_ between treatment and control groups was very nearly statistically significant (*p* = 0.06), and if true, would be of physiological relevance. It is likely that ICP amplifies the effects of many hours of change in the balance between CSF production and outflow resistance. Therefore, what may seem to be minor differences in the outflow resistance rates between groups may result in quite significant differences when measured over a longer time scale.

Our results suggest that the involvement of CSF outflow resistance as a mechanism for ICP elevation cannot be discarded, however it is important to note that it may not be the only mechanism responsible for this pressure rise post-stroke. CSF production is the other compartment of CSF dynamics that influences the volume of CSF in the craniospinal compartment at any given time. An indirect estimation of CSF production rates by others could not detect an increase in CSF production post-stroke [13]. Therefore, it is possible that hypothermia treatment reduces resistance to CSF outflow but may also act on CSF production mechanisms to further reduce the rate of production post-stroke. Reduced CSF production rates have been demonstrated with lower body temperatures [22,23,24], however this has not been examined in the context of ischaemic stroke. It is also not yet known whether the effect is sustained following rewarming. One of the remarkable attributes of the prevention of ICP rise with hypothermia treatment in these studies is that it is seen many hours after rewarming, indicating a longer term, switch-like mechanism of body cooling. Hypothermia may therefore have a synergistic effect on CSF dynamics, altering both outflow resistance and production mechanisms to cause an overall reduction in CSF volume ultimately preventing ICP elevation.

Another possible mechanism of interest relates to the glymphatic system. Glymphatic circulation drives CSF into the brain along periarterial spaces and drives interstitial fluid out [25]. This fluid may exit via perivenous spaces but there is also evidence to suggest the involvement of intramural periarterial drainage (IPAD) [26,27]. It is likely that IPAD plays an important role in the clearance of large molecules, however its contribution to the clearance of water and ions remains unknown. While the mechanisms of CSF clearance do not pertain to this present study, glymphatic dysfunction has been reported in stroke [28], and its contribution could therefore be important if glymphatic circulation is found to play a significant role in CSF outflow.

It is also possible that hypothermia may prevent post-stroke ICP rise by mechanisms unrelated to CSF dynamics. A recent study by Kalisvaart et al. [29] suggests that tissue compliance may be an important adaptive response against elevated ICP. The study assessed neuronal volume and morphology in several brain regions following stroke-induced brain injury in rats and demonstrated that widespread tissue and cell shrinkage occurs 24 h post-stroke in brain areas that were outside of the injury site. These findings may have important implications regarding the mechanistic actions of hypothermia to prevent ICP elevation post-stroke. Perhaps tissue compliance may be further altered following hypothermia treatment, and it is this cell volume reduction that aids in buffering elevated ICP.

We have demonstrated that gradual cooling successfully prevents ICP elevation, reduces infarct volume post-stroke, and has a role in reducing CSF outflow resistance. A limitation of this study relates to the animal variability in the CSF outflow analysis. Another limitation of this study is the choice of anaesthetic used. Previous studies have reported that anaesthesia can alter responses in CSF flow [25] and have suggested the use of ketamine/xylazine as the preferred choice in studies investigating CSF [30]. Isoflurane anaesthesia was used here in order to match the design of previous studies investigating CSF outflow resistance and ICP [6,13]. Moreover, the long duration of the experimental procedure made isoflurane a more convenient option than other alternatives. Another limitation relates to the time point of CSF outflow resistance measurements. Our time point was chosen to enable us to confirm ICP elevation in normothermia animals. However, outflow resistance would need to be increased for some time, and at constant CSF production rates, before ICP begins to rise. By the time of peak ICP at 24 h, outflow resistance presumably has already renormalized. So, the optimal time point for infusion of aCSF to measure outflow resistance is likely earlier than the 18 h post-stroke time point used in this study. Future work should therefore be done in the absence of significant ICP elevation in both groups, which should provide a more informed investigation into the relationship between hypothermia, CSF outflow resistance, and ICP.

## 5. Conclusions

In conclusion, our results suggest that hypothermia treatment has important effects on CSF volume dynamics. CSF outflow resistance has been implicated as a mechanism for ICP elevation following experimental stroke. Moreover, hypothermia treatment has routinely been shown to prevent this ICP rise, suggesting hypothermic cerebroprotection acts directly on this mechanism. Having a mechanistic understanding of how hypothermia prevents ICP elevation is crucial if the treatment is to be translated to stroke patients. Therefore, these results are the first steps towards elucidating the mechanism involved in intracranial pressure elevation and hypothermia treatment following experimental stroke.

## Data Availability

Data available upon reasonable request.
